# Predictive Stability of Aggregation in Glycoconjugate Vaccines Using Advanced Kinetics Modeling and High-Throughput Screening

**DOI:** 10.3390/pharmaceutics18050564

**Published:** 2026-04-30

**Authors:** Tao Ju Cui, Didier Clénet, Martinus A. H. Capelle, Milena Opacic

**Affiliations:** 1 Johnson & Johnson, 2333 CN Leiden, The Netherlands; mcapelle@its.jnj.com (M.A.H.C.); mopacic@its.jnj.com (M.O.); 2 Global Bioprocess Development, Vaccine CMC Development & Supply, Sanofi, 69280 Marcy l’Etoile, France; didier.clenet@sanofi.com

**Keywords:** stability, biopharmaceuticals, vaccines, modeling

## Abstract

**Background:** Predictive modeling of vaccine stability is an essential tool for accelerating development and ensuring product quality, particularly when long-term data are limited. To ensure high-quality input for accurate stability predictions, it is often necessary to allocate substantial analytical resources. **Methods:** This study demonstrates that integrating high-throughput screening (HTS) techniques such as ultraviolet–visible spectrophotometry (UV-VIS), intrinsic fluorescence and dynamic light scattering (DLS) with Advanced Kinetics Modeling (AKM) results in a synergistic approach, facilitating the development of robust predictive stability models. **Results**: Here, we applied AKM to a glycoconjugate vaccine targeting extra-intestinal pathogenic *Escherichia coli* (ExPEC). The aggregation processes observed via size-exclusion high-performance liquid chromatography (SE-HPLC), DLS, turbidity by UV-Vis absorbance and local changes in tryptophan microenvironment captured by intrinsic fluorescence were effectively described by the developed kinetic models. **Conclusions**: Using accelerated stability data across multiple temperatures, AKM successfully described key degradation pathways. Furthermore, the HTS assay results showed strong correlation with SE-HPLC data, indicating that these assays provide an efficient alternative requiring minimal analytical resources, material, and time.

## 1. Introduction

Shelf-life, product robustness and stability are critical parameters in the pharmaceutical industry, as they underpin the quality, safety, and efficacy of products throughout their development and commercial lifecycle. Biological products are often susceptible to thermal degradation, necessitating the determination of appropriate storage conditions. To substantiate product stability or immunogenicity/efficacy, stability studies are typically conducted at the intended shelf-life temperature as well as at elevated temperatures to monitor relevant changes in quality attributes over time. Given that biologics are often expected to have shelf-lives spanning several years, these stability assessments represent a significant bottleneck in the drug development process.

Consequently, there is substantial interest in predictive approaches that allow the estimation of shelf-life from short-term stability data. Importantly, the advantages of predictive stability modeling extend beyond reducing development timelines; they also encompass improved understanding of stability mechanisms, support for temperature excursion evaluations, comparability across manufacturing sites, and optimized formulation development. Moreover, the ability to model degradation pathways can provide deeper insights into the mechanisms underlying product instability.

While traditional methods continue to be suitable in many cases, oftentimes, biomolecules have more complex non-linear reaction pathways. Shelf-life estimation methods, as described in the current International Conference on Harmonisation (ICH) Q1 guidelines, were designed more than two decades ago with small molecules in mind. Simple linear regression is mainly proposed to fit experimental data obtained at a single storage temperature. Such a linear model can fail to adequately describe the complex stability behavior of bioproducts, which involve complex and multi-step reactions. This key issue can be overcome using Advanced Kinetics Modeling (AKM), in considering both simple linear and more sophisticated non-linear profiles.

In several published studies [1,2,3,4], kinetics-based models developed using stability data at different temperatures are found to provide a significantly more accurate approximation when compared to the linear regression models of stability data at only one temperature, as typically used in classical stability modeling (based on ICH Q1E). Resultantly, the shelf-life of some products could be assessed more accurately based on limited data if a kinetic model is applied than if a linear regression model is used for stability evaluation [3,5,6,7,8,9].

The latest draft ICH Q1 guideline recognizes the framework for predictive stability, including AKM, as a mature and viable tool to support initial and extended shelf-life [10]. One such approach, AKM, considers multiple reaction mechanisms simultaneously or in parallel and identifies the most appropriate model based on statistical criteria such as the Bayesian Information Criterion (BIC) and the Akaike Information Criterion (AIC) [6]. AKM has been successfully applied to characterize the degradation kinetics of various vaccines, polypeptides, and therapeutic antibodies [2,3,9,11,12] and to predict the stability of various glycoconjugate vaccines [1,13,14].

In this study, we demonstrate the application of AKM to a nine-valent glycoconjugate vaccine targeting extra-intestinal pathogenic *Escherichia coli* (ExPEC). ExPEC is a virulent pathogen that can cause urinary tract infections as well as other severe infections, such as meningitis and sepsis [15]. While these conditions can normally be treated with antibiotics, the presence of and rise in antibiotic-resistant genes is making ExPEC-related cases increasingly difficult to treat [15,16,17]. This trend has been linked to an increase in hospitalizations, deaths, and healthcare costs; in the United States alone, an estimated 80,000 deaths could potentially be linked to *E. coli* sepsis [18]. An effective vaccine product against ExPEC is therefore urgently needed, and efforts were made to develop a Phase 3 nine-valent O-antigen polysaccharide glycoconjugate vaccine, which consists of polysaccharide antigens covalently linked to a carrier protein: a detoxified form of exotoxin A derived from *P. aeruginosa* [19,20].

During development, manufacturing sites were changed, and to assess in a quick yet accurate manner whether these changes had any impact on product quality, an initial assessment was performed using predictive stability modeling. Furthermore, glycoconjugate vaccines are inherently complex and prone to degradation [21], including aggregation and structural changes in both the polysaccharide and protein components. By employing AKM, we were not only able to model a chemical process such as deacetylation, but—using high-throughput screening (HTS) methods—were also able to capture more challenging autocatalytic types of reaction processes, including aggregation and structural changes observed via shifts in intrinsic fluorescence signal. In summary, we demonstrated that the AKM method, in conjunction with HTS, can be a powerful tool for predicting degradation kinetics of critical quality attributes (CQAs) specific to glycoconjugate vaccines.

## 2. Materials and Methods

### 2.1. Study Design

As it was not known beforehand which CQAs were suitable for predictive stability modeling, an investigation following good modeling practices for accelerated stability studies was conducted to determine which temperatures and timescales were relevant and which CQAs were stability-indicating and suitable for use with AKM [22]. Several attributes were studied using the methods described below (Table 1). An accelerated stability study was set up, generating data for up to three months at temperatures of 5 °C, 25 °C, 40 °C, and 45 °C. The latter temperature was selected in order to remain at least 5 °C below the $T_{m}$ of the glycoconjugate protein (55 °C) while still providing a meaningful increment above 40 °C.

### 2.2. ExPEC9V Vaccine

The components are as follows: O-antigen polysaccharides of the extra-intestinal pathogenic *E. coli* (Eco) serotypes O1A, O2, O4, O6A, O15, O16, O18A, O25B, and O75, separately bioconjugated to the carrier protein, and a genetically detoxified form of exotoxin A (dEPA) derived from *Pseudomonas aeruginosa*. The O-antigen polysaccharides have an isolectric point (pI) of 5.5. The drug substance was manufactured at Johnson & Johnson (Bern, Switzerland). The drug product was manufactured at Sanofi (Marcy-L’Etoile, France).

### 2.3. Methods

#### 2.3.1. Enzymatic-Colorimetric Assay

To quantify the level of O-acetylation in ExPEC glycoproteins such as EcoO16 and EcoO25B, the drug product (DP) is precipitated using acetone and salt as follows [23]: First, 600 µL of ExPEC DP sample is transferred into a 5 mL Eppendorf tube. Then, 1 µL of saturated sodium chloride solution was added, and the tube was briefly vortexed; 2400 µL of acetone (<−18 °C) was added, and the tube was briefly vortex again. The sample was then incubated for 1 h (2–8 °C) in the fridge, followed by centrifugation at 9800× *g* for 10 min at 3 °C. The supernatant was removed and washed with acetone, centrifuged again and then dried at RT. The ester bonds were subsequently hydrolyzed under alkaline conditions in the precipitated sample by adding 90 µL of 0.1 M sodium hydroxide to the pellet. After neutralization with 0.1 M chloride hydroxide, the samples were filtered through a molecular weight cut-off (MWCO) membrane filter, and the filtrate—containing the released acetate—was quantified by an enzymatic-colorimetric reaction on a microtiter plate.

#### 2.3.2. SE-HPLC

Size-exclusion chromatography was used to separate the mono-, di-, and tri-glycosylated forms in the sample matrix using an isocratic gradient of PBS 1× mobile phase. A Supelco TSKgel G3000SWXL column, 5 µm, 7.8 × 300 mm (Sigma, St. Louis, MO, USA), was used in combination with an HPLC system equipped with a Alliance UV detector (Waters, Milford, MA, USA). Prior to analysis, the HPLC system lines were primed by flowing Milli-Q water for 10 min at a flow rate of 10 mL/min, followed by flushing the lines for 20 min at a reduced flow rate of 1 mL/min. For sample injections, samples were diluted to a final concentration of 60 µg/mL in 1× Tris-buffered saline (TBS) directly in HPLC vials. Each analysis consisted of two injections, with an injection volume of 150 µL per run at a flow rate of 0.6 mL/min. The mobile phase consisted of 1× phosphate-buffered saline (PBS), which was filtered using a “Rapid”-Filtermax 0.22 µm polyethersulfone (PES) filter (mobile phase A) and Milli-Q water (mobile phase B). Data were analyzed using Empower 3 to determine glycosylation populations.

#### 2.3.3. High-Throughput Screening Assays

To provide a holistic picture of degradation, high-throughput screening (HTS) assays were used in addition to the standard CQAs. These included UV–visible (Vis) absorbance, intrinsic fluorescence, and dynamic light scattering (DLS).

DLS measurements were performed using a DynaPro Plate Reader II (Wyatt, Goleta, CA, USA). A volume of 100 µL of undiluted sample was dispensed in triplicate into half-area UV-transparent 96-well plates (UV-Star, Greiner, Kremsmünster, Austria). For each well, five acquisitions of 5 s at 25 °C were recorded. The cumulant method was used to estimate the average hydrodynamic radius of the particles. Data were analyzed using Dynamics V7.5.0.17.

For absorbance and intrinsic fluorescence measurements, samples were pipetted into a 96-well plate and checked for the absence of bubbles or droplets using a flatbed scanner. The plate was then loaded into a BioTek Synergy Neo2 plate reader (Agilent, Santa Clara, CA, USA) and equilibrated before measurement. For UV-Vis absorbance, wavelengths between 250 and 500 nm were used. The background signal between 350 and 500 nm was used for scattering correction by fitting

$$s=a+\frac{b}{\lambda^{c}}$$

to the background data. This correction spectrum was subsequently extrapolated and subtracted from the 250–500 nm absorbance profile [24]. For intrinsic fluorescence, an excitation wavelength of 280 nm was used, and emission spectra between 300 and 450 nm were recorded.

#### 2.3.4. Modeling Background

Many reactions are possible from a chemical standpoint, especially for larger macromolecular complexes. These can be captured using a phenomenological differential equation as a function of α, which describes the reaction progress over time, from α=0 (no reaction) to α=1 (complete reaction). The truncated Šesták–Berggren equation below is used as a general reaction model:

(1) $$\frac{d\alpha }{dt}=k\left(T\right)f\left(\alpha \right)=A\times \exp\left(-\frac{E_{a}}{RT}\right)\times {\left(1-\alpha \right)}^{n}\times \alpha^{m}$$

Here, A is the pre-exponential factor, $E_{a}$ is the activation energy, R is the universal gas constant, T is the temperature in Kelvin, and n and m represent the reaction order and the autocatalytic contribution.

In the absence of an autocatalytic component, m=0. If n=0 or n=1, the equation is reduced to simple linear or first-order kinetics, respectively. Consecutive multistep reactions can be modeled by adding multiple right-hand-side terms of Equation (1), each with its own parameters ($A_{i},E_{a,i},\;n_{i},\;m_{i}$, etc.). In this study, we limited the model to two sub-reactions (i=1,2) with $m_{1}=0$.

#### 2.3.5. Model Solution and Calculation of Prediction Intervals

The kinetic parameters in Equation (1) were fitted to the 3-month dataset by non-linear least-squares using the lmfit (v1.3.4) library in Python v3.12. This resulted in many models, each with different order parameters. If multiple models are suitable for a given problem, the application of Akaike and Bayesian Information Criteria (AIC & BIC) helps balance between the goodness of the fit of the experimental results by the prediction curves, the number of required models and the number of parameters used. The “just right” model is selected considering not only the quality of fit (such as the sum of residual squares—RSS) but also the number of data points and model parameters [25]. The weighted Akaike Information Criterion (wAIC) and the Bayesian Information Criterion (wBIC) were computed for each model, and models were ranked and selected based on these weights to obtain realistic predictions [25,26].

Prediction intervals were calculated by adding random noise to the initial data set to account for assay variability, similar to what was done in previous studies [3,9,25,26]. This procedure was repeated 199 times to generate a total of 200 bootstraps. Long-term predictions from these simulations were saved in an array from which the 2.5th and 97.5th percentiles were extracted to derive the 95% confidence intervals. The prediction interval was then calculated following [26]:

$$\mathrm{prediction}\;\mathrm{width}=\sqrt{{\left(\mathrm{confidence}\;\mathrm{width}\right)}^{2}+{\left(t_{\frac{\alpha }{2},n-p}\times RMSE\right)}^{2}}$$

where the upper or lower prediction widths are derived separately from the percentiles (thus resulting in asymmetric prediction intervals). Here, n is the number of data points and p is the number of parameters.

#### 2.3.6. Use of Generative AI (GenAI) During the Writing Process

Microsoft 365 Copilot was used in this article for grammar, spelling, and sentence improvements. After using this tool, the authors reviewed and edited the content as needed.

## 3. Results

For the multivalent glycoconjugate DP vaccine against ExPEC, several CQAs were monitored at 5 °C, 25 °C, 40 °C, and 45 °C for a period of up to 3 months. The temperature of 45 °C is well below the measured $T_{m}$ of 55 °C.

### 3.1. O-Acetylation

The multivalent vaccine is composed of nine different O-antigens, among which the rhamnose OH groups in antigens O16 and O25 are partially acetylated. Previous studies have shown that reduced acetylation may lead to decreased immunogenicity, a pattern observed in other bacterial pathogens [27,28,29,30,31]. As such, O-acetylation was designated as a relevant quality attribute. Because the product is stored in a liquid state, both temperature and time are expected to promote the loss of O-acetyl groups. Monitoring O-acetylation is therefore critical to ensuring vaccine stability and efficacy.

**Figure 1:**
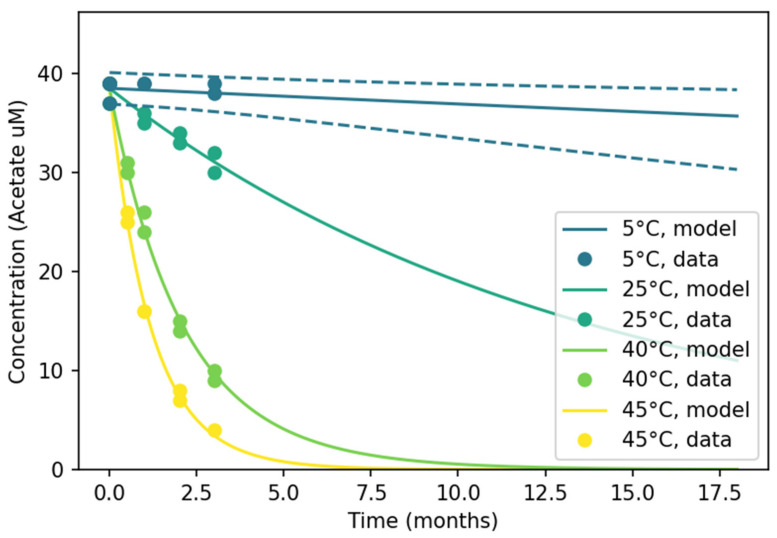
Acetate concentration of ExPEC9V plotted vs. thermal stress duration in months. The colors indicate different temperatures (5 °C, 25 °C, 40 °C and 45 °C), whereas the solid lines indicate the kinetic model predictions for a given temperature. The blue dashed lines indicate the 95% prediction interval (PI).

Figure 1 presents data from an enzymatic colorimetry assay. AKM assessed one-step and two-step degradation kinetics of different orders, concluding that a one-step Arrhenius model best describes the O-acetyl moiety loss. This finding aligns with previous results for another glycoconjugate vaccine [1].

Although validation data for extended time points were not available in this instance, the model’s low RMSE indicates good accuracy, consistent with other datasets in the literature modeled using AKM [1,2,9,11,13].

### 3.2. Aggregation Kinetics

Having established the importance of monitoring O-acetylation as a marker of stability and its susceptibility to temperature- and time-dependent degradation, attention next turns to another critical aspect of vaccine quality: aggregation kinetics. Understanding how the multivalent vaccine responds to thermal stress at the molecular level requires a multifaceted analytical approach, as described below.

**Figure 2:**
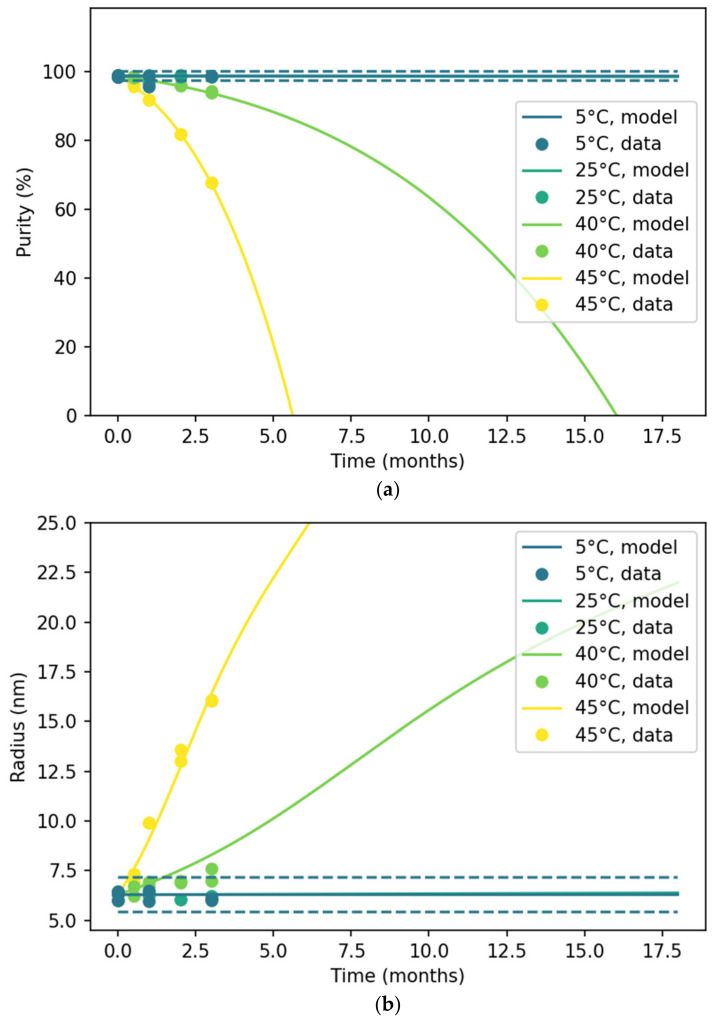
(**a**) Glycosylated concentration of ExPEC9V DP plotted for four different temperatures (5 °C, 25 °C, 40 °C and 45 °C) against time. The solid lines indicate the model predictions, and the dashed lines for 5 °C model indicate the 95% PI. (**b**) The hydrodynamic radius acquired through DLS is plotted against four different temperatures (5 °C, 25 °C, 40 °C and 45 °C) and time. The dashed line for the 5 °C model indicates the 95% PI.

Multiple analytical assays were employed to monitor the aggregation kinetics of the multivalent vaccine at the intended storage temperature of 5 °C and at elevated temperatures (Figure 2). The SE-HPLC data (top) demonstrated minimal changes in glycosylated product concentration at 5 °C and 25 °C. In contrast, a rapid decline was observed at 40 °C and 45 °C, indicative of accelerated degradation. The autocatalytic-type parameter m, derived from the two-step kinetic model, was found to be greater than zero, supporting the presence of autocatalytic processes.

Complementary results from DLS obtained using high-throughput assays corroborated these findings, showing an accelerating trend in particle size increase consistent with SE-HPLC data. Specifically, DLS measurements revealed negligible changes in hydrodynamic radius at 5 °C and 25 °C, while—similar to previous observations—marked size increases occurred at 40 °C and 45 °C, further confirming temperature-dependent aggregation behaviors.

To gain a more comprehensive understanding of aggregation phenomena, it is essential to complement size-based analytical assays with orthogonal high-throughput techniques. In this context, spectroscopy-based methods provide valuable insights by characterizing protein aggregation from distinct molecular perspectives. Here, UV-Vis absorbance and intrinsic fluorescence were measured for all samples.

**Figure 3:**
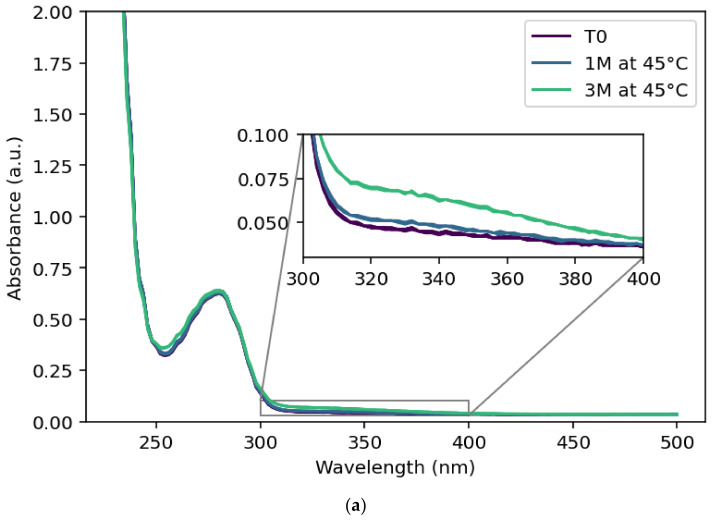

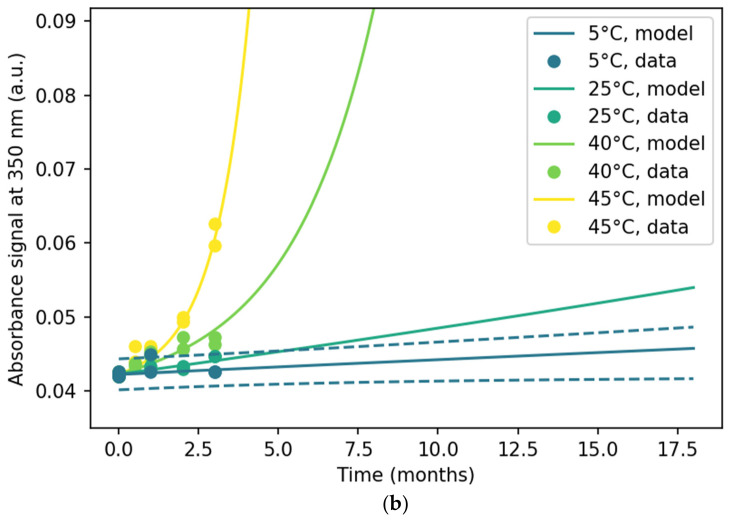
(**a**) The UV-Vis absorbance signal plotted for unstressed (T0, dark purple) sample, 1M at 45 °C sample (blue) and 3M at 45 °C (teal). (**b**) The absorbance value at 350 nm wavelength plotted against time for different temperatures (colored dots). The AKM fitted model is indicated by the colored line. The dashed line indicates the 95% PI.

Protein aggregation was assessed by measuring turbidity at 350 nm (Figure 3), as most proteins do not absorb at this wavelength [24]. Plotting turbidity at 350 nm over time showed an accelerating trend consistent with DLS and SE-HPLC results. Aggregation is a type of physical degradation that can arise from multiple causes, including adsorption to surfaces, denaturation, or precipitation [32]. To better understand the origin of the observed aggregation, intrinsic fluorescence was recorded between 300 and 450 nm after excitation at 280 nm. The amino acids tryptophan (Trp), tyrosine, and phenylalanine act as fluorophores and provide additional information on protein conformation. Of these, tryptophan is especially sensitive: a Trp residue buried in the protein core experiences a less polar environment than one exposed on the surface. Upon partial unfolding, previously buried residues can become solvent-exposed, serving as intrinsic probes of unfolding. This manifests as a red-shift in the signal and a decreased fluorescence intensity.

### 3.3. Intrinsic Fluorescence

**Figure 4:**
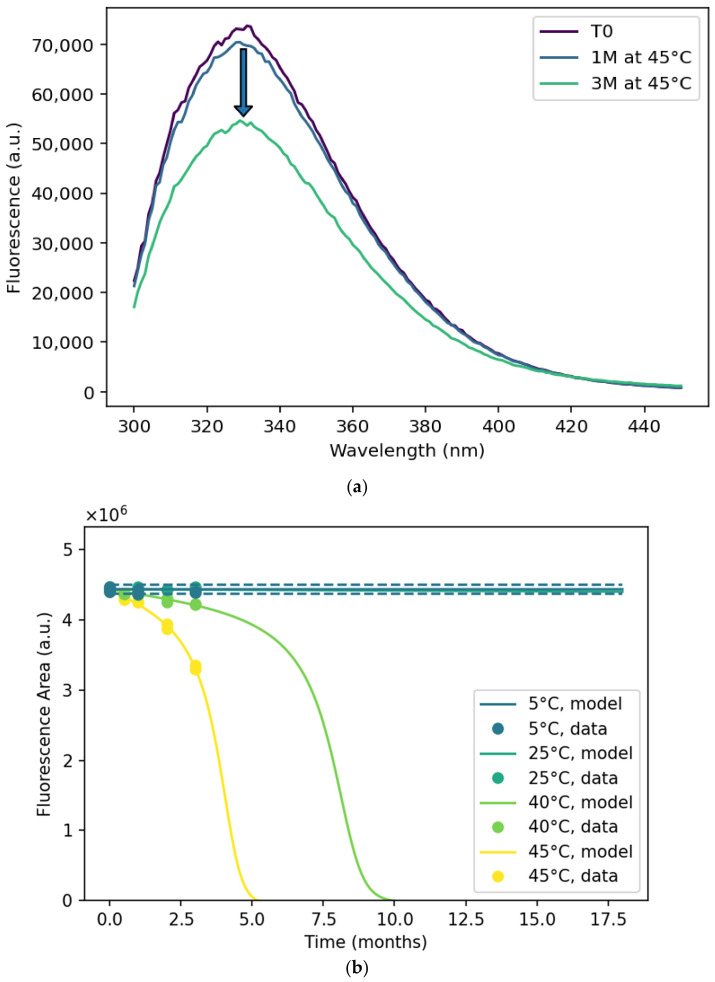

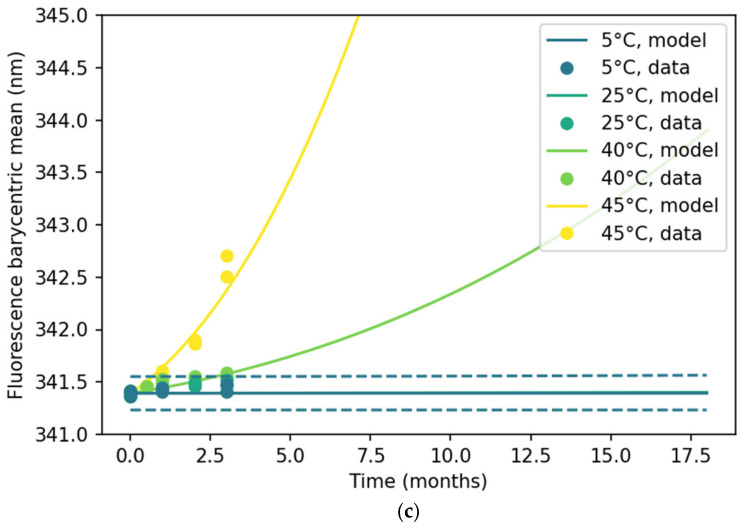
(**a**) Fluorescence spectrum of unstressed (T0, dark purple), 1 month at 45 °C (blue line) and 3 months at 45 °C (green line) due to excitation at 280 nm. (**b**) The area under the curve of individual samples is plotted for different temperatures (colors) and durations. The solid lines indicate the model outputs at the different temperatures. The dashed lines indicate the 95% PI for the 5 °C model. (**c**) The fluorescence barycentric mean plotted for different temperatures (colors) and durations. The dashed lines indicate the 95% PI for the 5 °C model.

The intrinsic fluorescence signal of ExPEC9V DP is shown in Figure 4a for an unstressed sample and two samples stressed for one and three months at 45 °C. At a glance, a decrease in maximum fluorescence intensity and surface area was observed when the DP was exposed to thermal stress. When the surface area is plotted against duration (Figure 4b), an overall accelerating, temperature-dependent trend is evident. To detect a possible red-shift in the signal, one could monitor only the peak intensity. However, because the signal is slightly noisy (Figure 4a), we utilized the barycentric mean fluorescence as a more robust alternative that accounts for the entire emission spectrum. In Figure 4c, the barycentric mean shows that at higher temperatures, the peak shifts toward longer wavelengths, indicating a minor red-shift.

Observed shifts in the fluorescence signal—primarily originating from Trp residues located within hydrophobic regions of the protein—could be suggestive of local unfolding events. Positions of Trp residues within the EPA protein are presented in Supplementary Figure S1. Exposure of additional hydrophobic residues may lead to the aggregation of partially unfolded glycoconjugates, which would then be detected by SE-HPLC, DLS, and turbidity assays.

In summary, the application of the AKM alongside high-throughput screening assays effectively elucidated the aggregation kinetics observed in this study. Notably, there was a strong (anti-)correlation between the high-throughput screening measurements and purity (%) obtained by SE-HPLC (Supplementary Figure S2). These findings indicate that high-throughput screening methods may serve as efficient and reliable surrogates for predicting protein stability and aggregation, offering the advantages of reduced material consumption and lower operational effort.

## 4. Discussion

Predictive stability modeling using advanced kinetics often requires substantial resources in both material and analytical effort, as a reliable model depends on high-quality data. Here, we show that the integration of Advanced Kinetics Modeling with high-throughput screening methodologies is highly effective for characterizing multiple degradation pathways in ExPEC9V, an investigational nine-valent glycoconjugate vaccine. The combined application of high-throughput assays and predictive modeling enables rapid assessment of stability profiles using reduced material and effort, which is advantageous both for early-phase development (e.g., formulation optimization), where material and resources are often scarce, and for late-stage development (e.g., shelf-life determination, specification setting, and comparability across manufacturing sites), where significant resources may still be required.

**Figure 5:**
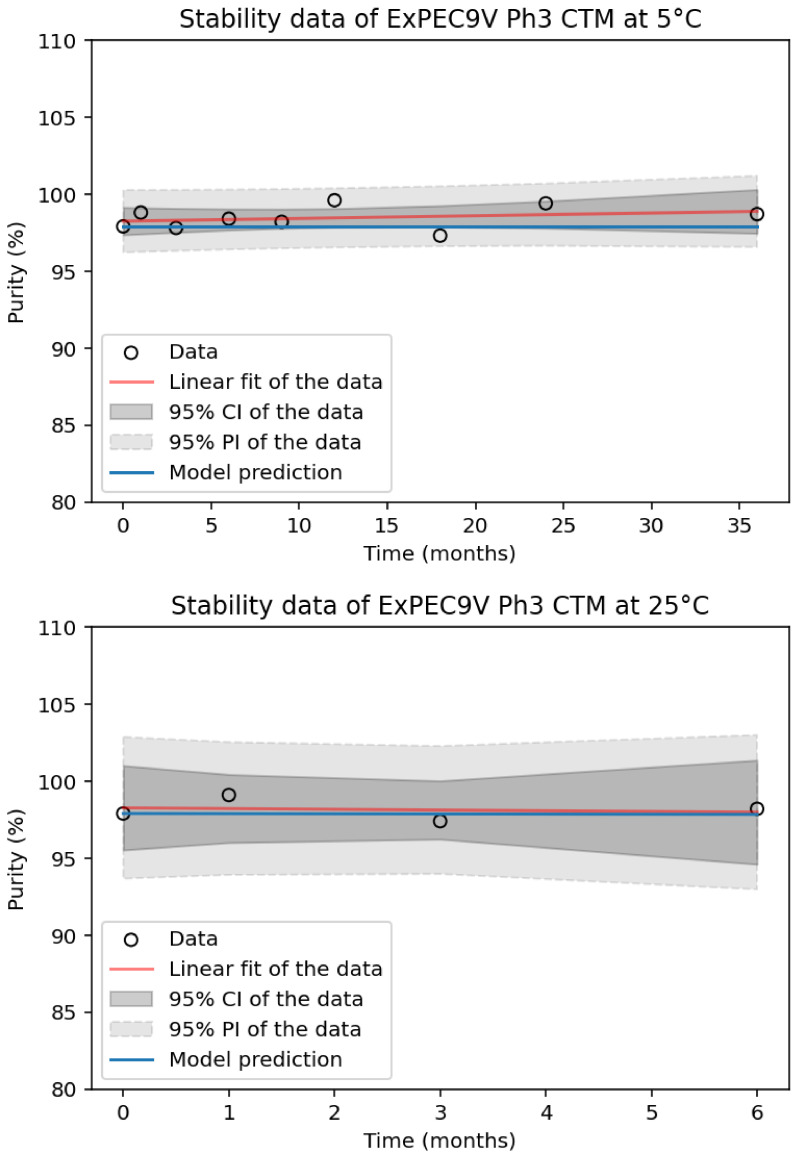
(**Top**): Long-term stability data (depicted as open black circles) for a single batch of Phase 3 clinical trial material (Ph3 CTM) of ExPEC9V drug product (DP) stored at 2–8 °C for up to 36 months are presented, superimposed with the Advanced Kinetics Model (AKM) prediction (blue line). (**Bottom**): Stability data at 25 °C for ExPEC9V DP up to 6 months are similarly overlaid with the AKM prediction (blue line). The red line represents a linear fit of the data, while the dark gray and light gray shaded regions correspond to the 95% confidence interval and 95% prediction interval, respectively.

To ensure a model’s reliability, it needs to be verified using long-term stability data points. A common approach for model verification involves constructing the model using short-term stability data, then employing long-term stability data points for verification [2,3,4,9,12,13]. This process determines whether the observed data fall within the 95% prediction intervals. Although direct long-term stability data for model verification are unavailable in this case, in-use and stability studies conducted on ExPEC9V drug product (DP) clinical trial material for Phase 3 demonstrated strong alignment with the AKM outputs regarding SE-HPLC purity. Specifically, no significant change in purity was observed for ExPEC9V DP stored for 36 months at 5 °C and for 6 months at 25 °C (Figure 5). Likewise, the results from controlled temperature cycling studies between 8 °C and 25 °C and between 25 °C and 40 °C agreed well with the predicted purity as determined by SE-HPLC (Appendix A.2). Taken altogether, the incorporation of additional stability data provides further confirmation of the model’s reliability and aligns with the predictive outcomes observed in this study.

In the study above, a single-step degradation model provided an accurate representation of O-acetyl moiety loss. Aggregation phenomena were identified through three orthogonal techniques—SE-HPLC, DLS, and turbidity measurements—with strong correlations observed among the methods, as indicated by the similar autocatalytic parameters extracted using the AKM. High-throughput screening assays also enabled protein stability modeling through intrinsic fluorescence measurements. The observed red-shifts in signal were successfully captured by AKM, indicating its potential to model chemical changes in the microenvironment of tryptophan residues and to describe potential unfolding events. Overall, the aggregation detection methods demonstrated a strong correlation (Supplementary Figure S2). Two-step kinetic models were applied to empirically describe the degradation/aggregation reaction. Here, n and m describe, respectively, the contributions from the decaying and accelerating (autocatalytic-type) factors in the kinetic equation [33]. In the context of kinetic modeling, the autocatalytic parameter *m* characterizes the lag phase observed prior to the onset of aggregation [22]. Specifically, when the reaction order *n* equals 1 and *m* is set to 1, Equation (1) is reduced to the well-known Prout–Tompkins equation, which is widely used to describe nucleation and aggregation phenomena [33,34].

Fitting with two-step AKM (with one autocatalytic term *m*) revealed that the autocatalytic parameter m was consistently greater than zero (Table A1), highlighting the significant role of autocatalytic-type processes during thermal degradation of these glycoconjugates. In particular, we observed in simulated degradation trajectories that m modulates the time-lag before the onset of aggregation both in single-step and two-step kinetics, with a larger value of m resulting in a longer time-lag (Supplementary Figure S3). It is important to note that, within the time points evaluated in this study, only limited degradation and aggregation of the protein were observed. Aggregation processes sometimes follow an S-shaped curve [34,35], and it is therefore possible that the later stages of the aggregation reaction—during which the rate may slow—have not yet been fully captured in the current kinetic equations or models.

To our knowledge, this study is the first to model these HTS-derived degradation kinetics using the AKM approach, highlighting the versatility of kinetic modeling and high-throughput screening for understanding glycoconjugate vaccine stability profiles. In principle, the methodology is broadly applicable and could be extended to other vaccine types beyond glycoconjugates. Such an approach enables rapid decision-making while enhancing the efficiency and reliability of vaccine stability assessments, thereby supporting the timely delivery of safe and effective vaccines.

## Figures and Tables

**Table 1:** Attributes examined through the following assays in this study.

Assay	Attribute
Enzymatic-colorimetric assay	O-Acetylation
SE-HPLC	Purity
DLS	Aggregation
UV-Vis Absorbance	Protein content, aggregation
Intrinsic fluorescence	Change in protein structure/conformation

**Figure A1:**
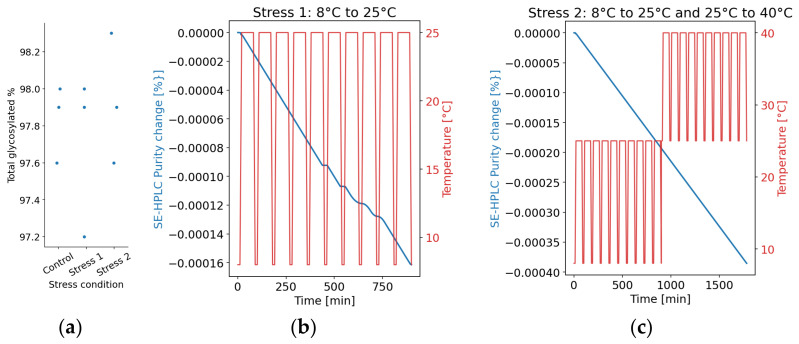
Temperature cycling results for ExPEC DP. (**a**) The glycosylated % results of unstressed samples (‘Control’) vs. samples that have undergone thermal stress profile 1 (‘Stress 1’) and stress profile 2 (‘Stress 2’). (**b**) Simulated temperature profile (red) of samples that have undergone stress profile 1 and the predicted change in glycosylated % (blue). (**c**) Simulated temperature profile (red) of samples that have undergone stress profile 2 and the predicted change in glycosylated % (blue).

## Data Availability

The original contributions presented in this study are included in the article/Supplementary Material. Further inquiries can be directed to the corresponding author.

## Supplementary Materials

The following supporting information can be downloaded at https://www.mdpi.com/article/10.3390/pharmaceutics18050564/s1. Figure S1. Crystal structure of Pseudomonas Aeruginosa Exotoxin A at 1.6A resolution (pdb: 1IKQ). Nine tryptophan residues are highlighted in stick-and-ball representation. Hydrophilic residues are highlighted in blue and hydrophobic residues are highlighted in red. Figure S2. Correlation plot illustrates the relationship between SE-HPLC purity results and high-throughput screening outcomes. Figure S3. Example of effect of varying autocatalytic parameter m on reaction process for a two-step degradation process. Here, the kinetic parameters of a given two-step degradation process are used.

## Abbreviations

The following abbreviations are used in this manuscript:

AIC	Akaike Information Criterion
AKM	Advanced Kinetics Modeling
BIC	Bayesian Information Criterion
CQA	Critical Quality Attribute
DLS	Dynamic Light Scattering
DP	Drug Product
HTS	High-Throughput Screening
ICH	International Council for Harmonisation
MWCO	Molecular Weight Cut-Off
PI	Prediction Interval
RMSE	Root Mean Squared Error
SE-HPLC	Size-Exclusion High-Performance Liquid Chromatography
UV-Vis	Ultraviolet–Visible Spectrophotometry

## Appendix A

### Appendix A.1

**Table A1:** Overview of fitted kinetic parameters for the main figures.

Figure + Assay	ln(A1 s) [−]	n1	Ea1 [kJ/mol]	ln(A2 s) [−]	Ea2 [kJ/mol]	n2	m2
1 Acetate	22.01	1.05	97.7	-	-	-	-
2A SE-HPLC	75.22	1	246.0	39.93	147.3	0	0.83
2B DLS	−1.92	1	242.0	79.06	248.7	2	0.46
3B Absorbance at 350 nm	−4.04	1	37.6	39.24	143.0	0	1.07
4B Fluorescence area under the curve	52.59	0	186.2	1.39	39.4	1	2.01
4C Fluorescence barycentric mean	36.39	1	153.6	41.02	137.9	1	1.73

### Appendix A.2

A temperature cycling study was conducted in an environmental climate chamber. Sample A is the unstressed sample and acts as reference. Sample B is the sample that underwent stress program 1, and sample C underwent both stress program 1 and stress program 2.

Stress program 1:
  Briefly, ExPEC9V DP samples of group DE were cycled
  Start at 8 °C
      # step1: 10 min hold at 8 °C
      # step2: 8 °C to 25 °C at 1.8 °C/min
      # step3: 1 h hold at 25 °C
      # step4: 25 °C to 8 °C at −1.8 °C/min
  This is repeated 10 times in total.

Stress program 2:
  Start at 8 °C
      # step1: 10 min hold at 8 °C
      # step2: 8 °C to 25 °C at 1.8 °C/min
      # step3: 1 h hold at 25 °C
      # step4: 25 °C to 8 °C at −1.8 °C/min
  This is repeated 10 times in total.

Subsequently, start at 25 °C
      # step1: 10 min hold at 25 °C
      # step2: 25 °C to 40 °C at 1.8 °C/min
      # step3: 1 h hold at 40 °C
      # step4: 40 °C to 25 °C at −1.8 °C/min
  This is repeated 10 times in total.

In both cases, a minimal effect of temperature degradation was expected according to the model, and this was also observed (Figure A1). Significant decreases in predicted glycosylation percentage are only observed following thermal cycling of the sample at elevated temperatures between 40 °C and 50 °C for extended periods (up to several days), which underscores the inherent stability of the product.
