# Supplementary material for: Predictive Stability of Aggregation in Glycoconjugate Vaccines Using Advanced Kinetics Modeling and High-Throughput Screening
Source: Pharmaceutics. 2026 Apr 30;18(5):564. doi: 10.3390/pharmaceutics18050564 (PMC13211225; doi:10.3390/pharmaceutics18050564)
Supplement: Supplementary file 1 [file pharmaceutics-18-00564-s001.zip › Supplementary Data.pdf]

Supplementary data

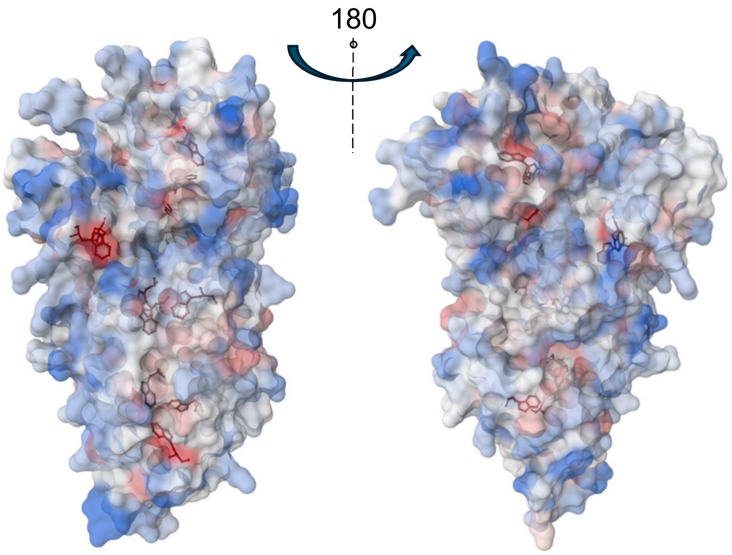

Supplementary Figure S1. Crystal structure of Pseudomonas Aeruginosa Exotoxin A at 1.6A resolution (pdb: 1IKQ). Nine tryptophan residues are highlighted in stick-and-ball representation. Hydrophilic residues are highlighted in blue and hydrophobic residues are highlighted in red.

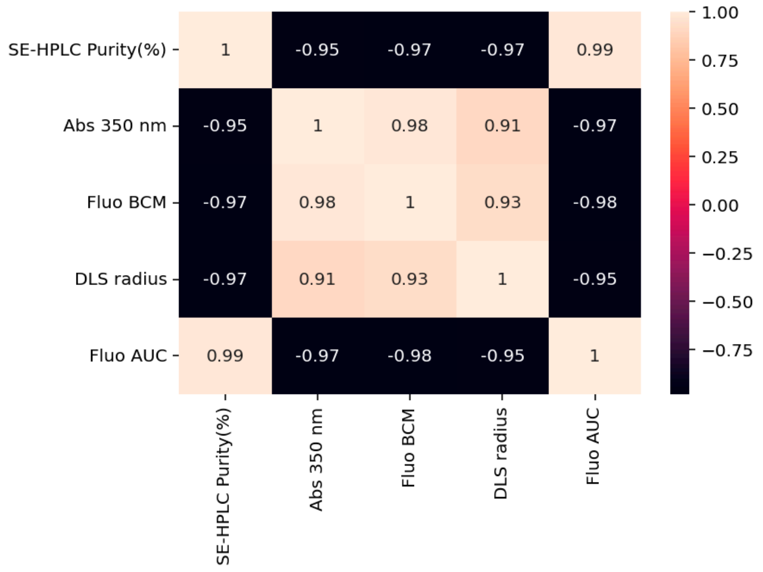

Supplementary Figure S2. Correlation plot illustrates the relationship between SE-HPLC purity results and high throughput screening outcomes.

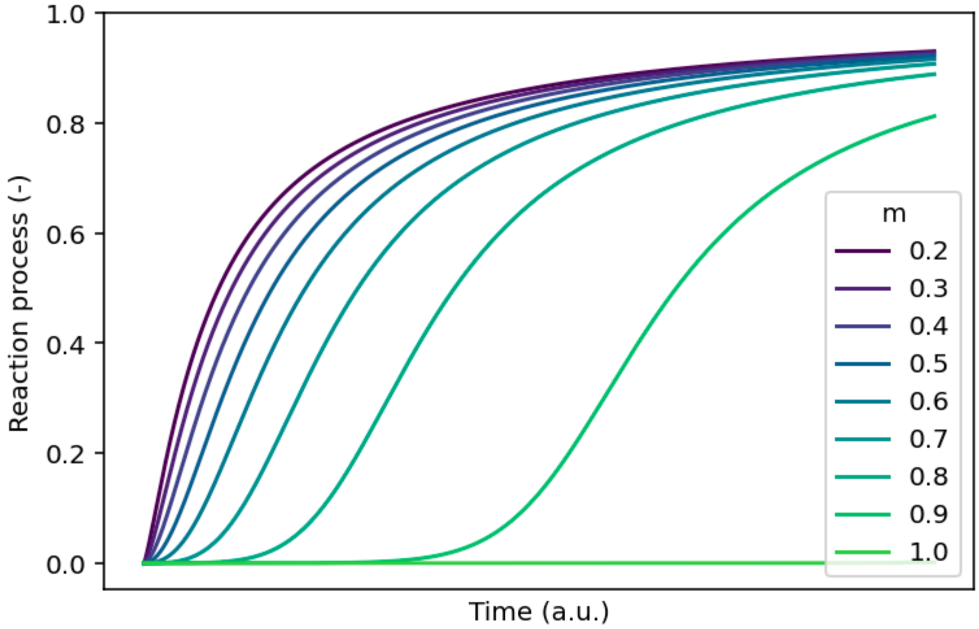

Supplementary Figure S3. Example of effect of varying autocatalytic parameter m on reaction process for a two-step degradation process. Here, the kinetic parameters of a given two-step degradation process are used.
